# Effects of Four Days Hiking on Postural Control

**DOI:** 10.1371/journal.pone.0123214

**Published:** 2015-04-22

**Authors:** Marcus Fraga Vieira, Ivan Silveira de Avelar, Maria Sebastiana Silva, Viviane Soares, Paula Hentschel Lobo da Costa

**Affiliations:** 1 Bioengineering and Biomechanics Laboratory, Universidade Federal de Goiás, Goiânia, Goiás, Brazil; 2 Department of Physical Education, Universidade Federal de São Carlos, São Carlos, São Paulo, Brazil; The University of Queensland, AUSTRALIA

## Abstract

Hiking is a demanding form of exercise that may cause delayed responses of the postural muscles and a loss of somatosensory information, particularly when repeatedly performed for several days. These effects may negatively influence the postural control of hikers. Therefore, the aim of this study was to investigate the effects of a four-day hike on postural control. Twenty-six adults of both sexes travelled 262 kilometers, stopping for lunch and resting in the early evening each day. Force platforms were used to collect center of pressure (COP) data at 100 Hz for 70 seconds before hiking started and immediately after arriving at the rest station each day. The COP time course data were analyzed according to global stabilometric descriptors, spectral analysis and structural descriptors using sway density curve (SDC) and stabilometric diffusion analysis (SDA). Significant increases were found for global variables in both the anterior-posterior and medial-lateral directions (COP sway area, COP total sway path, COP mean velocity, COP root mean square value and COP range). In the spectral analysis, only the 80% power frequency (F80) in the anterior-posterior direction showed a significant increase, reflecting the increase of the sway frequencies. The SDC revealed a significant increase in the mean distance between peaks (MD) and a significant decrease in the mean peak amplitudes (MP), suggesting that a larger torque amplitude is required for stabilization and that the postural stability is reduced. The SDA revealed a decrease in the long-term slope (Hl) and increases in the short-term (Ks) and the long-term (Kl) intercepts. We considered the likelihood that the presence of local and general fatigue, pain and related neuromuscular adaptations and somatosensory deficits may have contributed to these postural responses. Together, these results demonstrated that four days of hiking increased sway frequencies and deteriorated postural control in the standing position.

## Introduction

Human postural control is actively controlled by the central nervous system (CNS) through the integration of sensory information from the visual, vestibular and somatosensory systems, which leads to reflexive and voluntary muscle responses [1, 2]. The role of the visual system in balance control has received much attention, but interest in the effects of prolonged exercise on postural control during bipedal quiet standing in both trained [3] and untrained [4, 5] subjects is growing, partly because prolonged exercise may be associated with the impairment of several sensory inputs. Moreover, localized calf muscle fatigue also alters postural control [6–8] and these muscles are largely recruited during prolonged walking.

Hiking is a widely practiced prolonged exercise activity. Depending on the distance covered, hiking can be considered a mild to an extremely exhausting activity. Fatigue and foot pain frequently appear during the first day and affect the performance until the end of the hike, especially on multi-day hikes.

Induced muscle fatigue has already been demonstrated to affect postural control in a standing position [9, 10]. Fatigue reduces the muscle’s ability to produce an expected force through a combination of physiological processes occurring at both the central and peripheral levels [11, 12] and decreases the functionality of proprioceptive muscle receptors [13]. The unreliable and distorted proprioceptive feedback produced by fatigued muscles, particularly the plantar flexors [10], results in an increased reliance on vision to cope with postural instabilities. Additionally, fatigue of the tibialis anterior muscle impairs the positional sensing of joints, resulting in reduced acuity of ankle positioning [13]. However, during long-distance running or walking, fatigue is not experimentally induced but is rather a natural effect of continuously exercising, and it may also decrease postural control.

After prolonged walking, distal muscle fatigue is believed to cause long-term walking pattern adaptations, as revealed by increased heel loading and decreased toe loading, which result in less effective dynamic foot function and are demonstrated by lowering of the plantar arch [14]. Subjects experiencing fatigue induced by prolonged walking first show local muscle fatigue at the m. tibialis anterior followed by instability of the gait rhythm, which is slowed to enhance local dynamic stability [15]. Moreover, the plantar pressure distribution changes and foot complaints observed after several days of walking likely reflect fatigue of the lower leg muscles and the avoidance of loading the most vulnerable parts of the foot [14].

In this regard, besides avoiding the overload of specific and painful foot structures, central organization of movement is modified as a fatigue compensating strategy [16, 17]. In the presence of fatigue, the motor system changes the interjoint coordination [17, 18], while movement trajectory and cycle duration remain invariant. These motor adaptations are evidences of compensating strategies [19] triggered by fatigue in order to avoid accumulated fatigue and to maintain a good motor performance.

Long-term hiking usually causes skin injuries and muscle, joint and foot pain, which lead to the transmission of inaccurate somatosensory information to the central nervous system (CNS) and may also affect postural control, similar to an effect that has been previously described in diabetic peripheral neuropathy and while standing on a foam surface [20, 21]. The group III/IV muscle afferents carrying pain information are activated and they alter the firing response of motoneurons [22]. The combination of these factors may changes how the subject moves [22] and increases the risk of trips, falls and injuries [23].

In central Brazil, a traditional hike occurs annually. For four days, hikers travel approximately 262 km, stopping for lunch and in the evening to sleep. The participants cover an average of 65 km per day under severe climatic conditions, including sun exposure, high mean temperatures of 32°C (range, 19°C to 35°C) and low relative air humidity (range 18% to 40%).

Thus, the aim of this study was to identify changes in the postural control of hikers during each of the four consecutive days of the hike, considering the severe conditions of this hiking trail. To the best of our knowledge, this issue has not been previously studied. We investigated postural control using different stabilometric descriptors to quantify not only sway but also the underlying physiological mechanisms related to fatigue and sensory impairment. Thus, global and structural stabilometric variables [24] were computed from force platforms signals such as reliable center of pressure (COP) descriptors [25], spectral descriptors (both global descriptors), and stabilometric parameters related to the sway density curve (SDC) [24, 26] and stabilometric diffusion analysis (SDA) [27] (both structural descriptors). SDC and SDA interpretation may offer more insights into the nature of the process controlling the standing posture. The global stabilometric descriptors do not consider the dynamic characteristics of COP displacement, which are less useful for understanding the mechanisms used by the CNS to control posture [10].

We sought to identify changes in stabilometric descriptors and assess the decreased functionality of postural stabilization mechanisms during hiking. We hypothesize that general and local fatigue, sensory impairments and pain experienced during the hike produce changes on postural stability as either effects or adaptation to fatigue and pain.

Part of this study has previously been presented as an abstract [28].

## Methods

### Subjects

The hike organizers require all participants to undergo a selective trial in which they must achieve minimal results to qualify for the four-day hike.

In 2013, seventy hikers enrolled in the selective trial. The hikers were selected after a two-day trial, during which they were required to walk at least 28.2 km within 3 hours and 10 minutes for male hikers or 3 hours and 20 minutes for female hikers each day. On the 1^st^ day, 20 hikers were eliminated; on the 2^nd^ day, 24 hikers were eliminated. The remaining 26 hikers (24 males, 2 females) that were able to complete the selective trial were invited and have agreed to participate in this study (32.6 ± 3.2 years-old, 78.4 ± 12.5 kg, 1.74 ± 0.23 m).

During the hike, the participants were accompanied by a support team comprising physicians, physiotherapists and physical education teachers who recorded all events, including injuries and medical interventions.

### Ethics Statement

The hikers underwent medical examinations and were fully informed of the procedures and the risks involved. They were allowed to stop the study at will and to refuse any of our tests.

The study was approved by the institutional ethics committee of the Universidade Federal de Goiás, Brazil (Approval Number 781/2013—Ethics Committee for Human Research, Universidade Federal de Goiás). All subjects provided written voluntary informed consent prior to participation. The experiment was conducted according to the Declaration of Helsinki.

### Hiking Characteristics

On the 1^st^, 2^nd^ and 4^th^ days of hiking, the road was relatively flat, and the temperature oscillated around the average for the dry winter season in central Brazil (32°C). The 3^rd^ day of hiking was the longest and covered the most challenging portion of the trail; the trail was a bumpy road with long uphill sections and a high temperature. The asphalt temperature was 47°C on the morning of the 3^rd^ day.

The hikers woke up at 4:30 am, began to walk at 5:30 am, and they stopped in the early evening at approximately 6:30 pm. The participants slept for 6.5 hours between 10:00 pm and 4:30 am each night of the hike. On the 1^st^, 2^nd^ and 3^rd^ days, the hikers rested for 45 minutes to eat lunch. The 4^th^ day was the shortest, and the hikers had 2 hours of rest during lunch and walked for only 3 hours in the afternoon.

### Experimental Procedure

Three identical AMTI force platforms (OR6-7 model) were prepared to collect ground reaction forces and moments to ensure that the data collection was performed immediately after the arrival of each participant. Thus, even when a small group of hikers arrived together, the data collection never took longer than 15 minutes because some authors have reported that the effect of acute exercise on postural control is no longer apparent 15 to 20 minutes after the exercising [29, 30].

During the four days of the hike, data were collected in the early evening (1^st^, 2^nd^, 3^rd^ and 4^th^ days), immediately after reaching the end of the trail, except on the 1^st^ day, when the data were also collected prior to the beginning of the hike (Pre-hiking), to establish a baseline for data analysis.

Each hiker executed three trials of bipedal standing with eyes open. During each trial, the subjects stood barefoot with the feet positioned at a comfortable distance from each other on the force platform for 70 s. The data were collected at 100 Hz. During the test, the subjects looked at a fixed eye-level target at a distance of 1.5 m, adjusted for the height of each subject. The subjects were instructed to stand with their arms alongside their body, to look straight ahead at the target and to maintain their position. A period of 30 s between trials was introduced to avoid a learning process [12, 31–33]. No familiarization session was conducted. When necessary, the order of the participants was chosen randomly each day.

### Data Analysis

The data were filtered using a 2^nd^ order zero-lag low-pass Butterworth filter, with a cut-off frequency of 12.5 Hz. The first 10 s of each acquisition were discarded to avoid transients [31–35], and the mean was removed by a detrending operation.

To compute features from the COP displacement, global stabilometric descriptors (total sway path—TSP, mean velocity—Velm, root mean square value—RMS, range and ellipse area), spectral analysis (mean power in the intervals 0–0.3, 0.3–1 and 1–3 Hz, Fmean, F50 and F80) and structural stabilometric descriptors (SDC and SDA) were employed. The calculations of each descriptor were applied for the signal in both the anterior-posterior (AP) and medial-lateral (ML) directions, except in the case of TSP and SDC.

For each day of hiking, the calculated values for each stabilometric descriptor were averaged from the three trials to improve the reliability [25].

To evaluate the short-term and long-term effects of hiking [14], the differences between the readings before the hike and at the end of the 1^st^ day (short-term effect) and at the end of the 4^th^ day (long-term effect) were calculated for each stabilometric descriptor.

A custom MATLAB code was written to process the data.

### Global Stabilometric Descriptors

The global stabilometric descriptors are summarized in Table 1. The RMS value and range allow for estimation of the overall postural performance, and the mean velocity has been suggested to represent the amount of activity required to maintain stability [36]. Higher range values indicate poor stability, whereas higher mean velocity values indicate an intense balancing activity [37].

**Table 1:** Global Stabilometric Descriptors Calculations.

	Short Name	Calculation
**Area**	Area	The elliptical area that encompasses 95% of the COP samples in 60 s, calculated using principal component analysis to estimate its axes, as proposed by Oliveira et al. [38]
**Root Mean Square**	RMS	$RMS=\sqrt{\frac{\sum_{i=1}^{N}{Cd(i)}^{2}}{N}}$
**Mean Velocity**	Velm	$Velm=\frac{fs}{N-1}\sum_{i=1}^{N-1}\left|Cd(i+1)-Cd(i)\right|$
**Range**	Range	*Range* = max(*Cd*)-min(*Cd*)
**Total Sway Path**	TSP	$TSP=\sum_{i=1}^{N-1}\sqrt{{\left(Cd_{ML}(i+1)-Cd_{ML}(i)\right)}^{2}+{\left(Cd_{AP}(i+1)-Cd_{AP}(i)\right)}^{2}}$

N is the number of samples, Cd is the COP displacement, and f_s_ is the sampling frequency. The RMS, Velm, and Range were calculated for both the AP and ML directions. The total sway path variable was calculated only for the resultant two-dimensional COP trajectory.

### Spectral Analysis

The power spectrum of the COP displacement obtained in each trial was estimated using the Welch method [39] with 2000 samples per periodogram, resulting in a spectral resolution of 0.05 Hz. The procedure also included using a Hann window, an overlap of 1000 samples and the subtraction of the best linear regression on each data window [31].

The mean power frequency (Fmean), given by Eq 1; the F50 (the median power frequency), which encompasses 50% of the area under the COP power spectrum; and the F80 power frequency, which encompasses 80% of the area under the COP power spectrum, were estimated:

$$Fmean=\frac{\sum_{i=1}^{N}P(i)F(i)}{\sum_{i=1}^{N}P(i)}$$ (1)

where P is the power, and F is the corresponding frequency of the COP power spectrum.

The average power spectrum obtained on each day of hiking from all 26 subjects (each of them computed from three trials) was calculated. The mean power for each day was compared at the intervals 0–0.3 Hz, 0.3–1 Hz and 1–3 Hz [40] because more than 90% of the total power of the COP signal was below 3 Hz [41, 42]. This analysis assumes that the low-frequency band is related to visual control, the middle-frequency band is related to vestibular and somatosensory information, and the high-frequency band is related to proprioceptive control and muscle activity [43–45]. A decrease in the mean power indicates an increase in postural stability [40]. Moreover, according to Baratto et al. [24], the low-frequency band of the COP spectrum (< 0.4 Hz) reflects the center of mass sway.

### Structural Stabilometric Descriptors

In addition to the global descriptors and spectral analysis, the COP data were evaluated according to structural stabilometric descriptors, including the SDC [26] and the SDA [27].

Briefly, the SDC is defined as the time-dependent curve that counts the number of consecutive COP samples falling within a circle with a 2.5-mm radius for each instant of time. The SDC analysis is an attempt to recognize anticipatory control strategies indicated by the COP signal by identifying data subsets within this signal and interpreting them as instants in which the anticipatory command is stable [24]. This method of analysis appears to be robust, particularly with respect to the radius used for the moving window, and is sensitive to the conditions of the subjects, including pathological conditions. The SDC was developed based on the qualitative aspects of motor control because this method is correlated with the control torques around the ankle joints[26].

Three SDC-related stabilometric parameters quantitatively describe the process of generating postural commands sequences: MP, the mean amplitude of the peaks, which is an estimate of the degree of the postural stability; MD, the mean distance between one peak and another, which corresponds to the amplitude of torque required for stabilization; and MT, the mean time interval between one peak and another, which is related to the rate of torque production [24, 26].

The MT parameter was normalized with respect to the sampling frequency, which provides a time dimension, and thus represents the mean time spent by the COP inside of the defined circle.

The SDA considers that the COP displacement resembles a fractal Brownian motion [46]. The signal is interpreted as a realization of a particular stochastic process. Collins and De Luca [46] described the stochastic properties of COP displacement through statistical mechanics parameters using a two-process, random-walk model. The features of the random walk are described by COP mean square displacements <Δr^2^> plotted in a log-log stabilogram diffusion plot (SDP). <Δr^2^> is computed by averaging the square distance between consecutive COP samples separated by *n*Δt time intervals, where *n* is an integer ranging from 1 to 1000, and Δt is the sampling period [27]. Assuming that the COP trajectory is a correlated random walk, the SDA reveals a short-term (s) process and a long-term process (l) described by the slope H and the intercept K of the best least-square lines in the corresponding regions of the experimental log-log SDP [27]. The short-term and long-term diffusion coefficients (Hs and Hl, respectively) and the corresponding intercepts (Ks and Kl, respectively) give the correlation between the COP displacement and the stochastic activity of postural control mechanisms for short and long time intervals [27].

When consecutive COP displacement values are positively correlated, the signal is said to show persistence (H > 0.5); when the values are negatively correlated, the signal is said to show anti-persistence (H < 0.5).

Increased Hs and Ks parameters indicate less precisely controlled movement, where the displacements are larger for a given time interval. Similarly, decreased Hl and increased Kl parameters are also indicative of less precisely controlled movement.

### Statistical Analysis

Because the data distribution for the global and SDA descriptors was Gaussian (Shapiro-Wilk test, p > 0.05), repeated measures analysis of variance (ANOVA) was applied each day to assess the effects of hiking. A post-hoc analysis was conducted with a Bonferroni correction applied.

However, because the data distributions for the spectral analysis and SDC descriptors were not Gaussian (Shapiro-Wilk test, p < 0.05), Friedman test design was applied. A post-hoc analysis using Wilcoxon signed-rank tests was conducted with a Bonferroni correction applied, in which the p value was divided by the number of compared pairs.

A paired T-test was applied to assess the differences between the short-term and the long-term effects of hiking.

A correlation analysis was performed to verify the redundancy in the results and to indicate the most relevant variables.

All statistical analyses were performed using SPSS software (v17, SPSS Inc., Chicago, IL), with the significance level set at p < 0.05.

## Results

Table 2 presents the characteristics of each day of the hike, including the distance traveled, the characteristics of the road and the temperature.

**Table 2:** Hiking Trail Characteristics on Each of the Four Days.

	1^st^ Day	2^nd^ Day	3^rd^ Day	4^th^ Day
**Route**	Goiânia/Itauçu	Itauçu/Goiás	Goiás/Faina	Faina/Araguapaz
**Distance (km)**	67	67	72	56
**Road Characteristics**	Flat	Flat	Uphill	Flat
**Temperature (°C)**	33	32	39	32

As registered by the support team, several hikers began to complain of foot injuries, which were mainly foot blisters, from the 2^nd^ day on. Muscle fatigue and pain also became evident, and several cases of muscle cramps that required medical assistance were registered. On the 3^rd^ day, three hikers asked to withdraw from the hike. These hikers were encouraged to continue and were closely monitored until the end of the day.

### Global Stabilometric Descriptors

**Fig 1:**
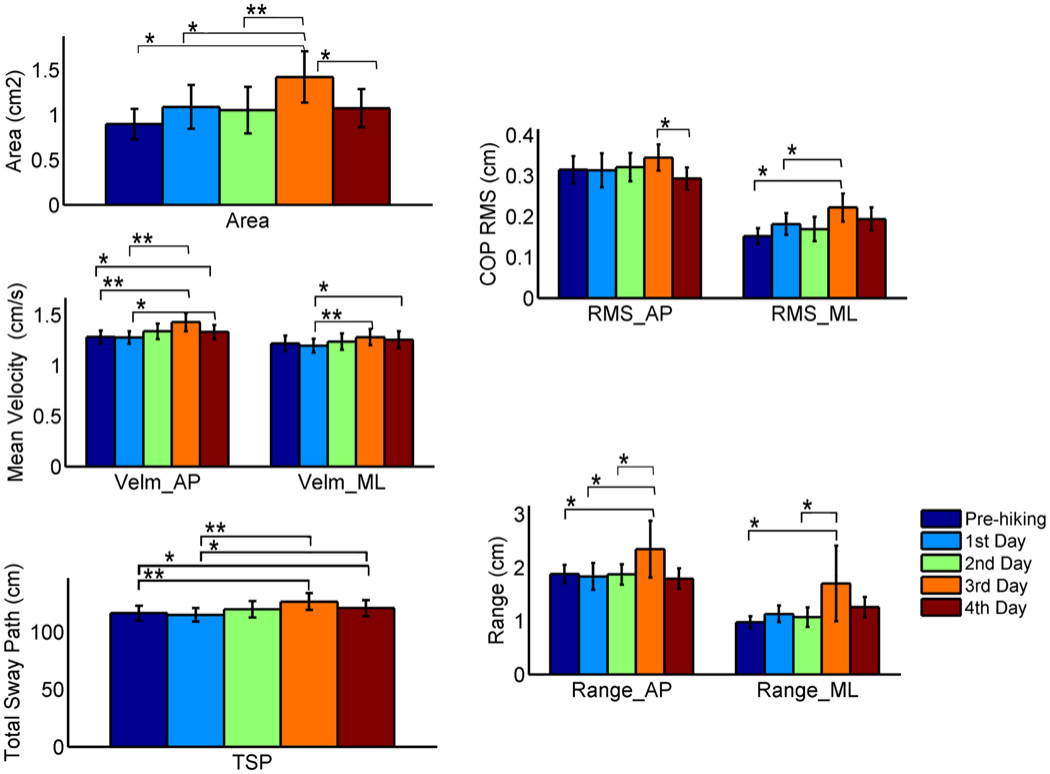
Global stabilometric descriptors. Significant pairwise comparisons: * p < 0.05, ** p < 0.001.

The results for the global stabilometric variables are summarized in Table 3. Fig 1 graphically presents the post-hoc tests among the hiking days.

**Table 3:** Global Stabilometric Descriptors.

	Pre-hiking	1^st^ Day	2^nd^ Day	3^rd^ Day	4^th^ Day	p-value
**Area (cm** ^**2**^ **)**	0.90 (0.42)	1.09 (0.60)	1.05 (0.64)	1.42 (0.71)	1.07 (0.52)	0.003*
**RMS AP (cm)**	0.31 (0.08)	0.31 (0.10)	0.32 (0.09)	0.34 (0.08)	0.29 (0.07)	0.014*
**RMS ML (cm)**	0.15 (0.05)	0.18 (0.07)	0.17 (0.07)	0.22 (0.09)	0.19 (0.07)	0.001*
**Velm AP (cm/s)**	1.28 (0.16)	1.28 (0.15)	1.34 (0.19)	1.43 (0.22)	1.33 (0.17)	0.001*
**Velm ML (cm/s)**	1.22 (0.19)	1.19 (0.17)	1.24 (0.20)	1.28 (0.20)	1.26 (0.21)	0.024*
**Range AP (cm)**	1.87(0.42)	1.83(0.61)	1.87(0.47)	2.34(1.31)	1.79(0.47)	0.012*
**Range ML (cm)**	0.97(0.28)	1.13(0.39)	1.07(0.45)	1.70(1.75)	1.26(0.48)	0.001*
**TSP (cm)**	116.2(15.7)	114.8(14.6)	119.6(17.5)	126.2(18.1)	120.6(17.5)	0.001*

Values expressed as the mean (standard deviation). p-value: repeated measures ANOVA.

* significant differences.

Significant differences were observed for all selected variables in both the AP and the ML directions (Table 3, last column—repeated measures ANOVA), particularly on the 3^rd^ day, indicating larger COP sways that are indicative of reduced postural stability.

The correlation analysis revealed strong and significant correlations between Area and RMS AP (r = 0.723 to 0.803, p < 0.001), Area and RMS ML (r = 0.815 to 0.943, p < 0.001), Area and Range AP (r = 0.710 to 0.741, p < 0.001), Area and Range ML (r = 0.797 to 0.899, p < 0.001), TSP and Velm AP (r = 0.933 to 0.973, p < 0.001), and TSP and Velm ML (r = 0.970 to 0.978, p < 0.001). A strong correlation between TSP and mean velocity was expected because the acquisition time was the same for all trials.

### Spectral Analysis

**Fig 2:**
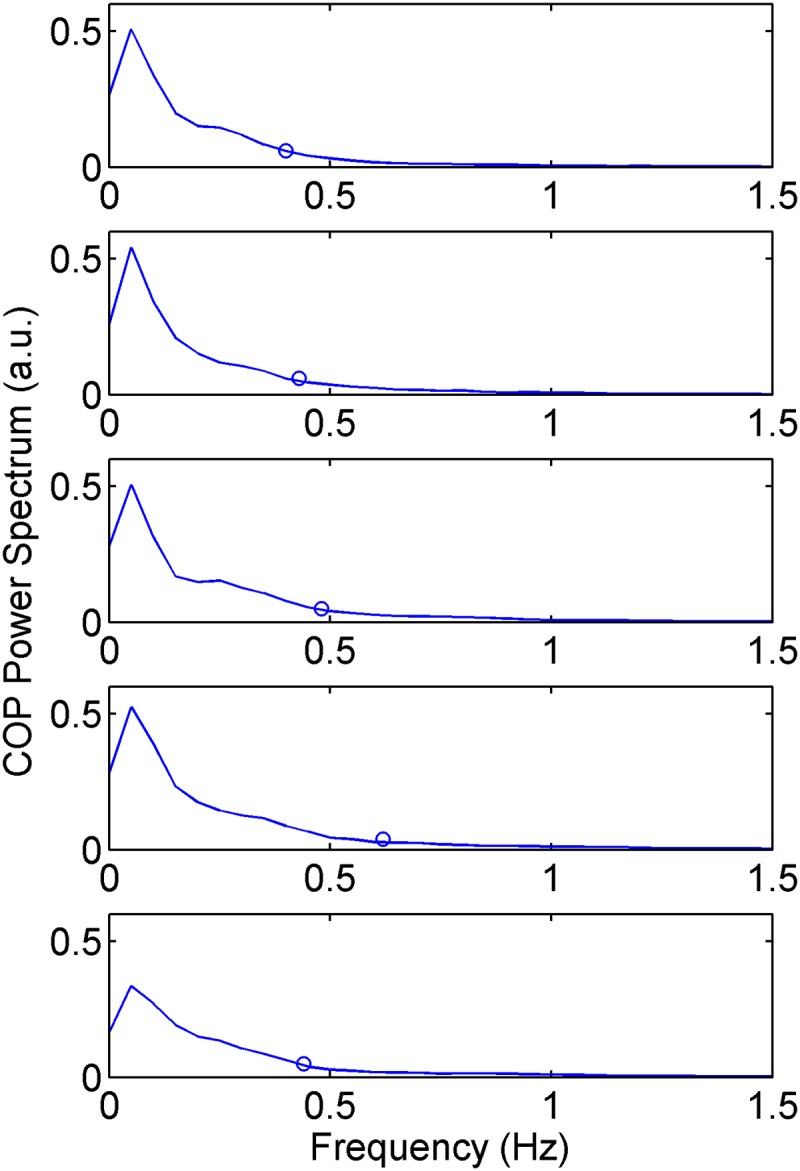
COP power spectrum and the F80 (circles) descriptor estimated for each data acquisition in the AP direction. The spectra plots are average estimates of 78 realizations for each day of the hike (3 trials x 26 hikers).

The results for the spectral analysis are summarized in Table 4. A significant difference was found for only the F80 AP variable (Table 4, last column—Friedman test), reflecting increased sway frequencies during the hike (Fig 2).

**Table 4:** Spectral Analysis.

	Pre-hiking	1^st^ Day	2^nd^ Day	3^rd^ Day	4^th^ Day	p-value
**Fmean AP (Hz)**	0.35(0.11)	0.36(0.14)	0.39(0.12)	0.47(0.39)	0.39(0.08)	0.241
**Fmean ML (Hz)**	0.80(0.31)	0.62(0.31)	0.76(0.33)	0.61(0.45)	0.63(0.28)	0.057
**F50 AP (Hz)**	0.19(0.07)	0.19(0.06)	0.21(0.07)	0.30(0.38)	0.21(0.04)	0.198
**F50 ML (Hz)**	0.35(0.16)	0.23(0.10)	0.29(0.13)	0.33(0.41)	0.26(0.12)	0.184
**F80 AP (Hz)**	0.40(0.11)	0.43(0.13)	0.48(0.13)	0.62(0.70)	0.44(0.10)	0.002*
**F80 ML (Hz)**	0.75(0.33)	0.60(0.42)	0.71(0.32)	0.64(0.77)	0.63(0.31)	0.602

Values expressed as the mean (standard deviation). p-value: Friedman test.

* significant differences.

The correlation analysis revealed weak or non-significant correlations between the F80 AP and any global, SDC or SDA variable.

**Fig 3:**
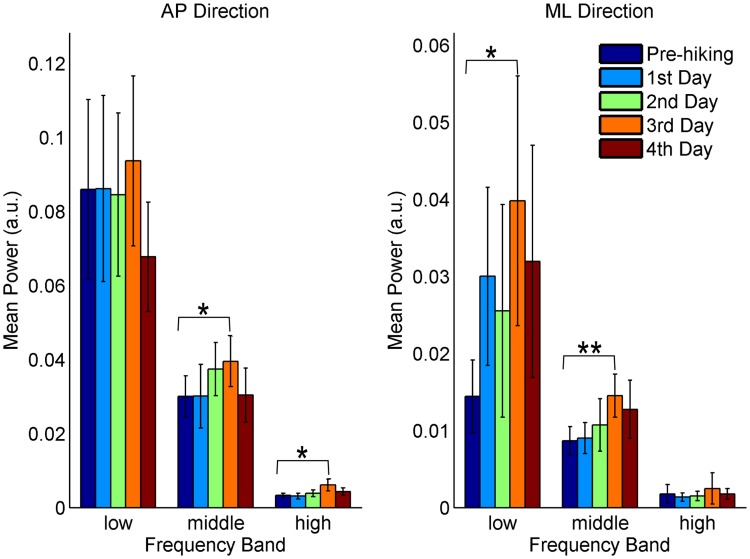
Mean power at different frequency bands for the hiking days. Significant pairwise comparisons: * p < 0.05, ** p < 0.001.

At the low-frequency band (0.1–0.3 Hz), there was only a significant increase in the mean power between the Pre-hiking measurements and the 3^rd^ day measurements (p = 0.03) in the ML direction (Fig 3).

At the middle-frequency band (0.3–1 Hz), there was a significant increase in the mean power between the Pre-hiking measurements and the 3^rd^ day measurements in both directions (p = 0.045 and p<0.001 in the AP and ML directions, respectively—Fig 3).

At the high-frequency band (1–3 Hz), there was only a significant increase in the mean power between the Pre-hiking measurements and 3^rd^ day measurements in the AP direction (p = 0.027—Fig 3).

The mean power in the ML direction was significantly lower than that in the AP direction at all frequency bands and for all data acquisitions.

### Structural Stabilometric Descriptors

The SDC analysis (Table 5) revealed a significant increase in the MD descriptor, and a significant decrease in the MP descriptor. Larger MD values and smaller MP values are both indicative of reduced postural stability.

**Table 5:** SDC Descriptors.

	Pre-hiking	1^st^ Day	2^nd^ Day	3^rd^ Day	4^th^ Day
**MT (s)**	0.58 (0.02)	0.58 (0.02)	0.58 (0.02)	0.58 (0.01)	0.58 (0.02)
**MD (mm)**	2.18 (0.55)^a^	2.22 (0.72)^b^	2.46 (0.74)	2.79 (0.56)^a^ ^,^ ^b^	2.44 (0.73)
**MP (mm)**	1.81 (0.55)^c^	1.96 (0.88)^d^	1.60 (0.55)^e^	1.31 (0.31)^c^ ^,^ ^d^ ^,^ ^e^	1.61 (0.64)

Values expressed as the mean (standard deviation).

Indexes—post-hoc tests, as follows:

^a, b, c, d^ p<0.001,

^e^ p = 0.03.

The results of the post-hoc tests are included in Table 5.

The resultant SDA (Table 6) revealed a significant difference for the Hl, Ks and Kl descriptors. The results of the post-hoc tests are included in Table 6.

**Table 6:** SDA Resultant Descriptors.

	Pre-hiking	1^st^ Day	2^nd^ Day	3^rd^ Day	4^th^ Day
**Hs**	0.59 (0.07)	0.58 (0.08)	0.60 (0.08)	0.62 (0.10)	0.60 (0.09)
**Hl**	0.13 (0.07)	0.16 (0.07)^a^	0.12 (0.07)	0.11 (0.07)	0.10 (0.06)^a^
**Ks**	1.27 (0.23)^b^	1.25 (0.26)^c^	1.34 (0.27)^d^	1.52 (0.22)^b^ ^,^ ^c^ ^,^ ^d^ ^,^ ^e^	1.35 (0.29)^e^
**Kl**	1.17 (0.19)^f^	1.16 (0.25)^g^	1.21 (0.23)^h^	1.36 (0.19)^f^ ^,^ ^g^ ^,^ ^h^	1.24 (0.23)

Values expressed as the mean (standard deviation).

Indexes—post-hoc tests, as follows:

^a, d, e, h^ p<0.05,

^b, c, f, g^ p<0.001.

Tables 7 and 8 show the SDA results for the ML and AP directions, respectively. Similarly, significant differences were found for the Hl, Ks and Kl descriptors in both directions.

**Table 7:** SDA ML Descriptors.

	Pre-hiking	1^st^ Day	2^nd^ Day	3^rd^ Day	4^th^ Day
**Hs**	0.50 (0.01)	0.50 (0.02)	0.50 (0.02)	0.52 (0.02)	0.52 (0.02)
**Hl**	0.11 (0.02)	0.18 (0.02)^a^	0.12 (0.01)	0.12 (0.02)^a^	0.12 (0.02)
**Ks**	0.63 (0.06)	0.59 (0.06)^b^	0.63 (0.07)	0.84 (0.06)^b^	0.72 (0.07)
**Kl**	0.43 (0.05)^c^	0.49 (0.05)^d^	0.49 (0.06)^e^	0.78 (0.06)^c^ ^,^ ^d^ ^,^ ^e^	0.61 (0.07)

Values expressed as the mean (standard deviation).

Indexes—post-hoc tests, as follows:

^a, b, e^ p<0.03,

^c, d^ p<0.001.

**Table 8:** SDA AP Descriptors.

	Pre-hiking	1^st^ Day	2^nd^ Day	3^rd^ Day	4^th^ Day
**Hs**	0.64 (0.01)	0.63 (0.02)	0.66 (0.01)	0.67 (0.02)	0.65 (0.02)
**Hl**	0.13 (0.02)	0.15 (0.01)^a^	0.12 (0.02)	0.11 (0.01)	0.09 (0.01)^a^
**Ks**	1.16 (0.05)^b^ ^,^ ^c^	1.14 (0.06)^d^ ^,^ ^e^	1.27 (0.05)^b^ ^,^ ^d^ ^,^ ^f^	1.41 (0.05)^c^ ^,^ ^e^ ^,^ ^f^ ^,^ ^g^	1.23 (0.06)^g^
**Kl**	1.07 (0.04)^h^	1.03 (0.06)^i^	1.09 (0.05)^j^	1.19 (0.04)^h^ ^,^ ^i^ ^,^ ^j^	1.09 (0.04)

Values expressed as the mean (standard deviation).

Indexes—post-hoc tests, as follows:

^a, b, d, f, g, h, i, j^ p<0.03,

^c, e^ p<0.001.

The changes observed for the SDA descriptors are indicative of reduced postural stability.

There was a strong correlation between MP and MD (r = 0.729 to 0.820, p < 0.001), as well as between Ks and Kl (r = 0.705 to 0.837, p < 0.001). Additionally, MD exhibited a stronger correlation with Area (r = 0.735 to 0.827, p < 0.001) than MP (r = -0.557 to -0.695, p = 0.001), and Kl also exhibited strong correlation with Area (r = 0.762 to 0.796, p < 0.001).

### Short-term and Long-term Effects

Significant differences between the short-term and long-term effects of hiking were found for the Velm AP (p = 0.001), Velm ML (p<0.001), TSP (p<0.001), AP Mean Power at the high-frequency band (p<0.001), ML Mean Power at the middle-frequency band (p = 0.048), and Hl (p<0.001) descriptors (Table 9).

**Table 9:** Short-term and Long-term Effects of Hiking.

	Short-tem	Long-term	p-value
**Velm AP (cm/s)**	-0.004 (0.074)	0.05 (0.07)	0.001*
**Velm Ml (cm/s)**	-0.02 (0.05)	0.04 (0.07)	<0.001*
**TSP (cm)**	1.43 (5.63)	4.36 (5.99)	<0.001*
**AP Mean Power high-frequency band (a.u.)**	-1.61e^-4^(1.07e^-3^)	1.01e^-3^(1.81e^-3^)	<0.001*
**ML Mean Power middle-frequency band (a.u.)**	3.78e^-4^(4.78e^-2^)	4.10e^-3^(9.20e^-3^)	0.048*
**Hl**	0.03 (0.09)^c^	-0.03 (0.08)	<0.001*

Values expressed as the mean (standard deviation). p-value: paired T-test.

* significant differences.

## Discussion

The aim of this study was to identify changes in the postural control of hikers during four days of hiking under severe climatic conditions by analyzing the COP sway. We investigated postural control using reliable stabilometric descriptors and interpreted our findings considering general fatigue, local fatigue, pain and sensory alterations.

### Reliability of Stabilometric Descriptors

The reliability of the COP global descriptors of postural steadiness was assessed by Lafond et al. [25] and Lin et al. [32]. Three 70-s quiet standing trials provided intraclass correlation coefficients higher than 0.8 for COP RMS, COP mean velocity, COP area and COP range but not for the frequency descriptors. However, the spectral analysis applied in this study reduced the COP power spectral density variance [39], which likely increased the F80 reliability [33]. The reliability of SDC parameters was appropriately analyzed by Barato et al. [24], whereas the reliability of SDA parameters was determined by Chiari et al. [27] by selecting appropriate boundaries for the short and long time interval regions in the log-log SDP. The reliability of the SDA parameters was also assessed by Amoud et al. [47]. By adopting reliable stabilometric descriptors, any differences observed during hiking would be attributable to local and general fatigue, pain and changes in sensory inputs rather than to fluctuations of these descriptors.

### Effects of General Fatigue

During the four-day hike under severe climatic conditions, significant changes in global stabilometric descriptors were found in both the AP and ML directions.

These results indicate an impairment in postural control, likely due to the negative effects of general fatigue such as physiological effects, the presence of metabolic products, [48] and the effects of sleep deprivation. Sleep deprivation appears to have only a minor effect, as observed by Degache et al. [49], but can influence the recovery of the physiological systems and, accordingly, can contribute to long-term effects.

Any muscular exercise that mobilizes a large part of the body, particularly over a prolonged time interval, induces physiological alterations with important mechanical impacts on the musculoskeletal system, that likely decrease the effectiveness of postural control mechanisms [48]. Following prolonged exercise, the muscle spindle shows impaired sensitivity in animals [50, 51], which may be due to the influence of metabolites or inflammatory substances [51] or to the modulation of reflex pathways [52].

When the level of lactate accumulation reaches its threshold during high-intensity exercise, the deterioration of postural control becomes significant, whereas during low-intensity exercise, postural control remains undisturbed [35, 53]. However, even at low intensities, prolonged, continuous exercise may disturb postural control [54], as was also observed in this study.

### Effects of Local Fatigue

In our study, four days of hiking appeared to cause fatigue of both the ankle plantar- and dorsiflexor muscles and the hip adductor and abductor muscles, as revealed by changes in the global and SDA descriptors in both the AP and ML directions. According to Gribble and Hertel [9], fatigue in all three lower limb joints contributed to postural control impairments in the sagittal plane (AP direction), whereas fatigue at the knee and hip joints led to postural control impairment in the frontal plane (ML direction). Additionally, Winter et al. [55] proposed that the ankle plantar- and dorsiflexors primarily control AP movements, whereas the hip adductors and abductors primarily control ML movements.

Prolonged hiking caused a significant increase in the power frequency of the COP signal only for the F80 AP descriptor, reflecting the increased sway frequencies in this direction (Fig 2). As the lower band of the COP spectrum primarily reflects the center of mass sways, changes in power toward higher frequencies are related to changes in the ankle torque pattern [24] because ankle stiffness increases according to the sway frequency [56]. These findings suggest that changes in the ankle torque pattern and ankle stiffness that occur during hiking are possibly caused by local fatigue in the muscles around the ankle joint.

The parameters extracted from the SDC analysis can be related to both anticipatory and postural stability control. Thus, a significant change (Table 5) in the MP variable reflects decreased stability of the postural control system during hiking. The SDC peaks are related to the active torque component, which is essential to increase ankle impedance in opposition to the toppling torque of gravity [26]. Thus, the decreased MP values during hiking indicates significantly decreased postural control activity related to the active torque component around the ankle, which is likely an effect of local fatigue.

The lack of a change in the MT descriptor value during hiking indicates that the shift from one postural command to another remains unaltered. Notably, considering the parameters related to the inter-peak timing (MT), if there is some type of phase lock between the sequence of the SDC peaks and the oscillation of the COM, there will likely be little or no dependence of the MT on experimental or pathological conditions [19].

A decreased MP value during hiking indicates decreased postural command stabilization, and an increased MD value indicates a larger amplitude of these postural commands, i.e., the amplitude of the torque required for stabilization is larger. Thus, the hikers have larger posturograms (see the Area descriptor in Table 3) during hiking because they generate larger and faster postural corrections. As previously discussed for the F80 AP descriptor, these alterations in torque production are likely due to local fatigue.

For the SDA descriptors, all Hs values were larger than 0.5, indicating persistence, whereas all Hl values were smaller than 0.5, indicating anti-persistence (Tables 6–8). The subjects became more unstable throughout the hike, with an increasing trend in Hs and a significant increase in Ks (Tables 6–8). A similar conclusion can be drawn from the significantly decreased Hl and increased Kl values during hiking, denoting poorer postural control. The significant increase in COP anti-persistence (decreased Hl and increased Kl) may have resulted from neural modulation of ankle torque to adjust tendon stiffness [33], which is related to local fatigue.

Therefore, the changes in postural control detected by structural stabilometric descriptors appear to be induced by increased muscle fatigue. The greater Hs and Ks values in the SDA indicate increased stochastic activity during short-term intervals. Muscle fatigue changes the capacity for fast and accurate contractions, and the neuromuscular system becomes unable to maintain constant and accurate muscle tension [57]. Compensation occurs in these fatigue states, which in turn results in larger COP displacements due to overcompensation [9].

Moreover, the increased postural sway observed could be caused by increased agonist-to-antagonist co-activation [58] and calf muscle synergist substitution [59]. Such strategies could be exaggerated to minimize fatigue effects and to provide the required amount of ankle torque to preserve postural stability, thereby increasing the frequency of the torque bursts [33]. In other words, the effects of fatigue appear to be compensated resulting in a new motor organization [17].

In general, the hikers were more stable in the ML direction than in the AP direction. This result was expected once the foot position on the force platform was not controlled. In a comfortable foot position, the COP sway in the ML direction is expected to be smaller than that in the AP direction because of the stance mechanics. Nevertheless, significant differences were found for the global descriptors (RMS ML, Velm ML, Range ML, Mean Power in the low- and middle-frequency bands) and the SDA ML descriptors (Hs, Kl and Ks) in the ML direction; thus, these differences could only be caused by the effects of hiking and not be compensated by a comfortable foot position [10].

### Effects of Sensory Impairments

Regarding the mean spectral power at the low-frequency band, there were significant differences between the Pre-Hiking measurements and the 3^rd^ day measurements only in the ML direction. According to Golomer et al. [43] and Oppenheim et al. [44], the low-frequency band is related to visual control. In support of this point of view, Saffer et al. [60] analyzed the coherence between muscle activity and body segment sway during quiet stance and found that closing the eyes led to a higher power of body sway at low frequencies and increased coherence for a number of segment-muscle relationships only at frequencies below 0.5 Hz.

Evidence suggests that head oscillations during walking exercise produces visual feedback deterioration and affects postural sway [61]; therefore, the significant difference found at the low-frequency band in the ML direction may be attributable to visual impairments.

However, visual feedback deterioration after walking exercise is more transient in the AP direction [55]. This could explain why no differences were found in the AP direction in this frequency band.

At the middle-frequency band, significant differences were found in both the AP and ML directions. As this band should relate to somatosensory and vestibular information [43, 44], these findings are possibly due to cutaneous and vestibular feedback impairments that affect both the AP and ML COP sway. These impairments include foot injuries such as those observed during the hike, particularly from the 2^nd^ day on, and hyperexcitation of vestibular inputs due to continuous body oscillation during prolonged walking.

At the high-frequency band, the significant difference in only the AP direction may be due to a greater influence of proprioceptive alterations induced by ankle muscle fatigue because this band is believed to relate to proprioceptive control and muscle activity [43, 44]. Fatigue causes negative effects on proprioception because it decreases muscular mechanoreceptor activation [62], specifically, fatigue of the tibialis anterior and triceps surae muscles decreases ankle proprioception [13].

### Effects of Pain

In addition to microruptures in muscle tissue during exercise, the sensation of muscle pain is provoked by a combination of metabolites found in muscle during exercise [63], as prolonged hiking. That metabolites act to activate sensory neurons that signal fatigue and muscle pain sensations [63]. The sensation of muscle fatigue precedes the sensation of pain [49], as observed during the hike, though not systematically registered by the support team.

At the light of new theories about pain and movement, our findings can be partially explained as adaptations to pain. According to Hodges and Tucker [22], the adaptations to pain involve redistribution of activity within and between muscles, modify the movement and stiffness altering the mechanical behavior and lead to protection of the painful part, avoiding further pain or injury.

All these aspects may have contributed to the observed changes in postural control and are complementary to the fatigue effects discussed earlier.

### Short-term and Long-term Effects

The variables Velm Ml, Velm AP, TSP, AP mean power at the high-frequency band, ML mean power at the middle-frequency band and Hl were significantly different between the short-term and long-term measurements, revealing a long-term effect of four days of hiking on postural control. During the hike, the hikers were exposed to a severe routine in which the recovery period of physiological functions was relatively short. The long-term effects could be observed even after the 4^th^ day, which involved the shortest hiking distance and the longest rest period during lunch.

However, the peaks observed in the variables on the 3^rd^ day were likely because this was the longest, most challenging and warmest day of the hike (Table 2). The hikers walked a 7.5% longer distance on the 3^rd^ day than on the 1^st^ and 2^nd^ days; additionally, the terrain was more demanding, and the temperature was higher. These three aspects are known to result in postural control alterations [48, 64].

### Correlation Analysis of the Descriptors

Global descriptors: According to the correlation analysis, Area, TSP and F80 AP appeared to be the most relevant global variables in this study. In fact, the other global variables provided the same information as Area and TSP. However, if it is important separately analyze the results in AP and ML directions, then the Velm AP and Velm ML should be considered because they are the most reliable measures of COP sway [25, 32].

SDC descriptors: The correlation analysis revealed a strong negative correlation between MP and MD, and MP exhibited a weaker correlation with Area. Therefore, compared to MD, MP appears to be more sensitive and less redundant to the changes in postural stability.

SDA descriptors: There was a strong correlation between Ks and Kl. Therefore, in this study, Hl and Kl were the SDA variables that best described the changes in postural stability.

In this study, all evaluated descriptors were found to be sensitive to changes in postural control after prolonged hiking. A different situation was observed when these descriptors were used to detect age-related changes in postural control [33], and not all of the descriptors were sensitive to aging. The correlation analysis indicated that Area, TSP, F80 AP, MP, Hl and Kl were the most sensitive (and least redundant) variables in this study.

### Limitations of the Study

There are several limitations in the present study. To guarantee that the data were collected before full recovery after effort [30], only opened eyes condition was assessed. Thus, establishing the precise relationship between visual feedback and muscle fatigue in postural control during long-term hiking is difficult in this study.

Moreover, as presented in Results session, pain-related complaints were quite frequent during the hike and required several interventions by the support team. Although these complaints certainly affected the somatosensory information and how the subjects moved, they were not systematically registered during the hike as proposed by Stolwijk et al. [14]. Therefore, carefully registering type, local and level of pain and fatigue experienced during the hike and relating them to the COP sway is of interest in subsequent studies.

## Conclusion

In conclusion, this study provides evidence that prolonged hiking induced significant impairments of the postural stabilization mechanisms, as detected by both global and structural COP sway descriptors in both the AP and ML directions, due to the effects of general fatigue, local fatigue, pain and sensory alterations.

## Supporting Information

S1 Data Collected Data. (ZIP)
